# Comparison of the Proximate Composition, Total Carotenoids and Total Polyphenol Content of Nine Orange-Fleshed Sweet Potato Varieties Grown in Bangladesh

**DOI:** 10.3390/foods5030064

**Published:** 2016-09-14

**Authors:** Mohammad Khairul Alam, Ziaul Hasan Rana, Sheikh Nazrul Islam

**Affiliations:** 1 Institute of Nutrition and Food Science, University of Dhaka, Dhaka 1000, Bangladesh; 2 Department of Nutritional Sciences, Texas Tech University, Lubbock, Texas 79409, USA

**Keywords:** orange-fleshed sweet potato, nutritional composition, total polyphenol content, total carotenoids content, Bangladesh

## Abstract

In an attempt to develop the food composition table for Bangladesh, the nutritional composition of nine varieties of orange-fleshed sweet potato was analyzed together with total carotenoids (TCC) and total polyphenol content (TPC). Each variety showed significant variation in different nutrient contents. The quantification of the TCC and TPC was done by spectrophotometric measurement, and the proximate composition was done by the AOAC method. The obtained results showed that total polyphenol content varied from 94.63 to 136.05 mg gallic acid equivalent (GAE)/100 g fresh weight. Among the selected sweet potatoes, Bangladesh Agricultural Research Institute (BARI) Sweet Potato 7 (SP7) contained the highest, whereas BARI SP6 contained the lowest amount of total polyphenol content. The obtained results also revealed that total carotenoids content ranged from 0.38 to 7.24 mg/100 g fresh weight. BARI SP8 showed the highest total carotenoids content, whereas BARI SP6 showed the lowest. Total carotenoids content was found to be higher in dark orange-colored flesh varieties than their light-colored counterparts. The results of the study indicated that selected sweet potato varieties are rich in protein and carbohydrate, low in fat, high in polyphenol and carotenoids and, thus, could be a good source of dietary antioxidants to prevent free radical damage, which leads to chronic diseases, and also to prevent vitamin A malnutrition.

## 1. Introduction

Sweet potatoes (*Ipomoea batatas*) are an exceptionally essential crop in several parts of the world, being produced in more than 100 countries. They are also a main food crop of the tropical and subtropical areas and, therefore, can provide a nutritional advantage to the people of rural and urban regions by enhancing their production and increasing consumption [1,2]. Sweet potato is positioned as the seventh most major food crop in the world, fourth in tropical countries [3] and the fifth most essential food crop on a fresh weight basis in developing countries after rice, wheat, maize and cassava, and it is cultivated in more than 100 developing countries [4]. It is the fourth most important crop in Bangladesh after rice, wheat and potato.

Malnutrition caused by the deficiency of vitamin A is known to be widespread among the rural people of developing countries, including Bangladesh. This deficiency causes children to succumb to common diseases and leads to the impairment of growth and development, vision impairment, poor immune functions and, in extreme cases, results in blindness and death [5].

In pregnant women, vitamin A deficiency (VAD) can have several consequences on pregnancy outcome, such as low birth weight of the baby, higher chance of transmitting HIV/AIDS virus infection and, in certain cases, may result in death of both the mother and child. Research findings concluded that vitamin A plays a vital role in the prevention of maternal mortality and can also prevent mother to fetus transmission of HIV/AIDS virus [5]. Roughly one million children have clinical signs and symptoms of VAD, and over 0.9 million children <6 years of age suffer from some degree of xerophthalmia; and 30,000 children become blind every year due to severe VAD in Bangladesh [5]. The high prevalence of micronutrient deficiencies is normally seen in the children of Bangladesh, particularly vitamin A, iron, iodine and zinc deficiency. The prevalence of night blindness among rural preschool-aged children in Bangladesh is 0.67% [5]. The VAD problem among the women of reproductive age is still considered to be a major problem. Over 2.7%, 2.4% and 2% of pregnant women, lactating women and non-pregnant/non-lactating women respectively are night blind in Bangladesh [5].

As most of the rural people cannot afford to buy animal products due to low income, there is a clear need to identify a potential alternative rich food source of vitamin A in terms of land/environmental sustainability, developing the rural economy and increasing the consumption to achieve the better nutritional status of the people of Bangladesh. Orange-fleshed sweet potato (OFSP) is relatively rich in β-carotene (precursor of vitamin A) and subsequently rich in vitamin A [6]. In addition, OFSP contains significant amounts of minerals (Fe, Zn, Mn), vitamins B and C, fiber and other micronutrients, such as polyphenols and carotenoids [6]. Polyphenols are widely known for their positive effect on human health. The inclusion of polyphenols in the diet provides protection against heart disease and prevents oxidation of LDL, by acting as ‘scavengers of free radicals’, especially peroxide, breaking its formation chain and neutralizing it [7]. Several authors have previously stated that the polyphenols in sweet potatoes exhibited antioxidative or free radical scavenging ability, boost humans’ defense mechanism against oxidative stress and, thus, inhibit the development of chronic diseases, like cancer, cardiovascular diseases, liver injury, etc. [7]. Like polyphenols and vitamin E, carotenoids also exhibit antioxidant activities [8].

To overcome the VAD problem, efforts were made to develop varieties that are high yielding, with medium-sized tubers and a high carotene content. Given these facts, the Tuber Crops Research Centre (TCRC), Bangladesh Agricultural Research Institute (BARI), developed seven high yielding varieties of sweet potato since 1988 through introduction, selection and hybridization [9]. There are now in total nine high-yielding varieties of OFSP in Bangladesh, and they are called BARI SP1, BARI SP2, BARI SP3, BARI SP4, BARI SP5, BARI SP6, BARI SP7, BARI SP8 and BARI SP9. Among the nine varieties of OFSP in Bangladesh, the highest yielding varieties are BARI SP8 and BARI SP9. The tuber yield potential of these two varieties varied between 40 and 45 tonnes per hector and is superior to the other seven varieties in Bangladesh.

Being rich in carotenoids, β-carotene and total polyphenol content (TPC), OFSP is gaining importance as the least expensive source of antioxidants, providing several essential previously mentioned health benefits. Consumption of OFSP roots can also provide sustainable vitamin A, which plays a major role in preventing and treating night blindness [10]. OFSP has substantial potential to contribute to a food-based approach to managing the problem of modern chronic diseases together with VAD, a major public health concern of the poor people of the world. Thus, there is a greater possibility that OFSP could be included in the normal diet of the consumer food habit to supplement as an alternative staple food source for the poor people in the age of widespread population growth and nutrition crisis. However, a significant proportion of consumers is not aware of the nutritive value of some high-yielding OFSP cultivars. Moreover, the biochemical constituent of OFSP varies among the varieties. Therefore, the assessment of the biochemical composition of different varieties is essential for selecting the cultivars having high amounts of the nutrients. In Bangladesh, no previous study was conducted on the nutritional content of different varieties of OFSP, and thus, the aim of the study was to estimate and compare the selected nutritional content of nine OFSP varieties produced in Bangladesh. This was the first comprehensive and systematic analysis of a large number of OFSP varieties in Bangladesh.

## 2. Materials and Methods

### 2.1. Reagents

Analytical-grade acetone, petroleum ether, n-hexane, dichloromethane, Folin-Ciocalteu reagent and acetic acid were purchased from Merck (Darmstadt, Germany). Gallic acid (PubChem CID: 370) was purchased from TIC (Japan).

### 2.2. Sample Collection and Preparation

The present study was conducted on nine varieties of OFSP grown in Bangladesh to estimate the proximate composition (moisture, ash, protein, fat, carbohydrate and fiber), TPC and total carotenoids content (TCC). The experimental samples were collected from Tuber Crops Research Centre (TCRC), Bangladesh Agricultural Research Institute (BARI), Gazipur, Bangladesh. All of the varieties were harvested in the same experimental field and received the same fertilizer and irrigation practice, whereas no information with regard to the growing conditions in the TCRC was available. The potatoes were harvested when they became mature at about four to five months; the average weight of each potato was 200 to 220 g. Six to ten freshly-produced samples of each potato variety were collected from the production field of TCRC of BARI. Table 1 represents the sample characteristics used in the study. All of the samples were collected as fresh as possible and processed at the Institute of Nutrition and Food Science (INFS) laboratory for analysis. Samples were gently washed with tap water immediately after collection to remove sand and other extraneous material before being washed with distilled water and then air dried. The samples were then cut into small pieces, placed in an auto seal bag and kept in a desiccator to inhibit moisture gain or loss. They were then ready for the determination of their proximate composition, TCC and TPC.

### 2.3. Proximate Composition

The proximate composition of the samples was carried out according to the AOAC official method for nutrient analysis [11]. Moisture content was determined by the oven method; ash content was determined by the dry ash method in a muffle furnace; protein content was determined by the Kjeldahl method (nitrogen content multiplied by 6.25); fat content was determined by using chloroform-methanol extraction; and crude fiber was estimated by the gravimetric method. Carbohydrate content was calculated using the following formula described by Rand et al. [12].

Carbohydrate content = 100 – {moisture (g%) + crude protein (g%) + total fat (g%) + crude fiber (g%) + total ash (g%)}; where (g%) = grams per 100 grams OFSP.

### 2.4. Analysis of Total Carotenoids Content

TCC in the sample was estimated by acetone-petroleum ether extraction followed by spectrophotometric measurement according to the modified Rodrigues-Amaya and Kimura method of total carotenoids analysis [13]. Extraction of carotenoid was performed by grinding of processed potato sample in a mortar and pestle, filtration through a sintered glass filter under vacuum and separation from acetone to petroleum ether. The petroleum eluent adjusted to a specific volume was read at 450 nm in a spectrophotometer (UV-1601, UV-Visible, Shimadzu, Tokyo, Japan) for the concentration of total carotenoids. Results were expressed as milligrams per 100 g of fresh weight (FW) (mg/100 FW).

### 2.5. Analysis of Total Polyphenol Content

The TPC in sweet potato extracts was estimated by the Folin-Ciocalteu colorimetric method according to Blainski et al. [14] with slight modification. Briefly, to appropriately-diluted sample extract (0.15 mL) was added 0.225 mL of 2-fold diluted Folin-Ciocalteu reagent, and this was kept for 5 min at room temperature. Then, 1.125 mL of 2% Na_2_CO_3_ solution were added, mixed well and kept for 15 min at room temperature. Finally, the absorbance was measured at 750 nm by a UV-VIS spectrophotometer (UV-1800, Shimadzu, Kyoto, Japan) and used to calculate TPC using a standard curve based on gallic acid. Results were expressed as milligrams of gallic acid equivalent (GAE) per 100 g FW (mg GAE/100 g FW).

### 2.6. Data Quality

The precision and accuracy were maintained by carrying out inter-laboratory analysis of TCC in the raw BARI SP2 between the INFS laboratory, the University of Dhaka and Nutritional the Biochemistry laboratory of the ICDDR, B (International Centre for Diarrheal Disease and Research, Bangladesh). No significant difference (*p* = 0.893) was found between the results (6.21 ± 1.35 mg per 100 g FW vs. 6.58 ± 1.12 mg per 100 g FW).

### 2.7. Statistical Analysis

The experiments were performed with three replicates for each sample of OFSP. Descriptive statistics were performed for all variables. One-way analysis of variance (ANOVA) was performed to test the differences among varieties for their proximate, total polyphenol and carotenoids contents. All analyses were performed with R software Version 3.2.2. The 5% level of least significance was used to determine any differences in the mean values between different sweet potato cultivars. Differences at *p* < 0.05 were considered to be statistically significant. Experimental results were expressed as the mean ± standard deviation (SD).

## 3. Results and Discussion

### 3.1. Proximate Composition

The moisture, ash, protein, fat, carbohydrate and fiber contents of the analyzed varieties of sweet potatoes are presented in Table 2.

#### 3.1.1. Color and Appearance

The flesh color of the sweet potatoes can be white, yellow or orange, and their skin color may either be white, yellow, orange, red or purple. The difference in the color of fruits and vegetables is due to the presence of different chloroplast pigments [15]. All of the sweet potato varieties selected in the study were light orange to orange in color (Table 1). The orange color is due to the presence of β-carotene [15].

#### 3.1.2. Moisture Content

From Table 2, it can be seen that the moisture content in the sweet potato varieties used in the study ranged between 70.95% ± 0.70% and 72.96% ± 0.15%. Wenkam [16] reported that fresh sweet potato had a moisture content of 77.8%. The variations in the moisture content among the sweet potato varieties can be due to the differences in the genetic composition and cultivation practices. In comparison with other roots and tubers, the sweet potato has a high moisture content and, thus, has a low dry matter content. The normal dry matter content is around 30%, but differs widely depending on aspects such as variety, geographic area, climate, amount of light, soil and cultivation practices [1]. Several authors have reported that the application of fertilizer significantly reduces the moisture content in the sweet potato [17]. The low moisture content signifies a high dry matter content and, thus, more carbohydrates and, consequently, a higher energy content [17].

#### 3.1.3. Protein Content

The protein content of the selected OFSP varieties ranged from 1.91% ± 1.01% to 5.83% ± 0.30%. The protein content was significantly higher in the BARI SP4 (5.83% ± 0.30%), while it was least in the BARI SP6 variety (1.91% ± 1.01%). Protein content in the diets of the low income population in developing countries like Bangladesh is derived mostly from foods of plant origin. The typical total protein content of sweet potato is as low as 1.5% FW and as high as 5% (dry weight (DW)). However, it is superior to other roots and tubers, such as cassava, and inferior to potato and cereals [1]. These tubers contain total protein from 1.0% to 2.5% (about 5% DW) [18,19]. Villareal et al. [20] found 2.8% protein in the sweet potato on an FW basis, while Senanayake et al. [21] found it in the range between 1.2% and 3.3% on a DW basis. This indicated that the OFSPs selected for the study, cultivated in Bangladesh, had higher protein content. Some other studies also found lower protein content than our study [22,23].

#### 3.1.4. Fat Content

Fat content of sweet potato varieties varied from 0.17% ± 0.10% to 0.63% ± 0.11% and showed significant (*p* < 0.05) differences among the varieties (Table 2). BARI SP5 showed an unusually higher content of fat (0.63% ± 0.11%) than the other varieties (*p* < 0.05). Like other roots and tubers, sweet potato is well recognized for its low-fat content. The results of fat content were similar to other study findings. Mu et al. [24] found 0.6% fat for sweet potatoes, while FAO [22] and Tumuhimbise et al. [23] reported the fat content of sweet potato to be around 0.2% and 0.17%, respectively. Ishida et al. [25] also found the fat content of sweet potatoes ranging from 0.2% to 0.33%.

#### 3.1.5. Total Carbohydrate

The result obtained in this study showed that carbohydrate content was between 21% and 25% in fresh samples of OFSP. Wenkam [16] in his study stated that fresh sweet potato contained 27% of carbohydrates, and FAO [22] reported 28% for fresh samples. Thus, we found less carbohydrate compared to these studies, and the reason could be factors like varieties and stages of maturity of the roots.

#### 3.1.6. Crude Fibers

In this study, dietary fiber ranged from 0.30% ± 0.11% in the BARI SP7 variety to the highest (0.54% ± 0.14%) in the BARI SP4 variety. In a study by Oomen and Grubben [26], the fiber content in fresh sweet potato was 3.9%. In 18 varieties of sweet potatoes in Hawaii, total fiber content ranged from 2.01 to 3.87 g/100 g FW [27]. FAO [22] reported 1.2%, and Ishida et al. [25] reported dietary fiber content of the sweet potatoes in the range between 2.28% and 11.7%. Compared to the above-mentioned studies, we found less fiber in our study, and this may be due to genetic and cultivation differences. However, Ingabire and Vasanthakaalam [28] found 0.11% to 0.14% in the Rwandan varieties in their study. On the other hand, based on dry weight, Oboh et al. [29] found that the crude fiber content of 49 sweet potato varieties ranged between 3.45% and 6.36%, and Senanayake et al. [21] found it in the range between 2.1% and 13.6 % in Sri Lankan varieties. Pectin, cellulose and hemicellulose and lignin are considered as dietary fiber [30]. Dietary fiber has recently received much importance, as it is believed to reduce the incidences of colon cancer, diabetes, heart disease and certain digestive diseases [28].

#### 3.1.7. Crude Ash

No significant differences were seen among the varieties in terms of ash content. The ash content of the OFSP varieties ranged from 1.17% ± 0.09% to 1.31% ± 0.04%. It must, however, be noted that BARI SP6 and BARI SP1 exhibited the same values of 1.17. Goodbody [31] reported that the total ash content in fresh sweet potato was 1.7%, and Ingabire and Vasanthakaalam [28] reported that the ash content in the fresh sweet potato tubers was between 0.40% and 0.44% in their study. Based on these amounts, we found less total ash in the sweet potato varieties, and there can be some factors that can have an influence on this low total ash content. One possible factor could be the use of fertilizer. Application of fertilizer together with sufficient irrigation can influence the nutrient content of OFSP, especially the mineral content [17]. Several authors have observed an increased concentration of minerals in the sweet potato leaves and roots with the increased application of fertilizer [32,33]. Besides fertilizer, the ash content of sweet potato varieties can also be influenced by other aspects, like soil, climatic conditions, etc. [34].

### 3.2. Total Carotenoids Content

Fruits and vegetables contain different types of carotenoids in different amounts [2]. Table 3 represents the TCC of the selected sweet potatoes. Among the selected sweet potatoes, BARI SP8 contained significantly higher amount of TCC (7.24 ± 0.19 mg/100 g FW) followed by BARI SP2 (6.38 ± 1.19 mg/100 g FW), BARI SP9 (4.61 ± 0.11 mg/100 g FW), BARI SP4 (3.72 ± 1.11 mg/100 g FW), BARI SP7 (3.01 ± 0.15 mg/100 g FW) and BARI SP5 (2.12 ± 0.28 mg/100 g FW). The other varieties contained comparatively lower amount of total carotenoids. Islam et al. [35] also reported similar results in their study. For some OFSP varieties, total carotenoid levels ranging from 0.390 to 8.823 mg/100 g have been reported [36]. In another study, OFSP varieties exhibited high amounts of carotenoids ranging between 7.91 and 12.85 mg/100 g [37]. OFSP varieties have been recognized as an excellent source of β-carotene. For instance, in Hawaii, 18 cultivars of OFSP were found to have 13.1 mg/100 g FW of β-carotene [27]. The β-carotene content in light yellow and purple flesh samples was in the range of 0.1 to 0.6 mg/100 g FW [15]. Thus, there is a scope for OFSP for increasing the vitamin A intake in Bangladesh. Sweet potatoes, carrots and leafy vegetables contain high levels of β-carotene, frequently greater than 8000 I.U./100 g, and can, therefore, meet the recommended daily intakes (5000 to 25,000 I.U.) [2].

As discussed in Section 3.1.1., there were samples that were light orange in color and thus, the BARI SP6 variety had lower total carotenoids content than BARI SP8, which was dark orange in flesh color. Because of the variations in flesh color, we observed variations in TCC, and this could also be true for β-carotene content in the varieties. The carotenoid content in OFSP can vary depending on cultivar and growing environment [38].

### 3.3. Total Polyphenol Content

The obtained result of TPC in nine varieties of OFSP is presented in Table 3. Among the selected sweet potatoes BARI SP7 significantly contained the highest amount of TPC (136.05 mg GAE/100 g FW), whereas BARI SP6 contained the lowest amount (94.63 mg GAE/100 g FW). Comparatively higher to lower TPC in OFSPs included in this study were as follows: BARI SP 7 > BARI SP 4 > BARI SP 5 > BARI SP 9 > BARI SP 2 > BARI SP 8 > BARI SP 3 > BARI SP 1 > BARI SP 6 (Table 3).

The phenolic contents of the hand-peeled OFSPs cultivated in the U.S. ranged from 0.06 to 0.23 mg GAE/g FW [15], whereas Truong et al. [39] reported 0.57 to 0.79 mg/g FW and Teow [40] found 0.03 to 0.95 mg/g FW. According to Poungmalee [41], TPC in an OFSP variety (T101) grown in Thailand was 1.94 mg GAE/g of fresh root sample. In this study, TPC of the OFSP cultivated in Bangladesh ranged from 0.95 to 1.37 mg of GAE/g FW of roots with peel. TPC content observed in our study is relatively lower than the value observed in Thai OFSP [41], while the value is higher than the U.S. OFSP [15,39,40].

Natural phenolic compounds have received increasing attention in the last few years, since a great amount of them can be found in plant and plant products, and thus, consumption of these products containing a greater level of such compounds may reduce the risk of the development of several diseases due to their antioxidant activity together with other health-promoting factors. The polyphenol content can vary among foods, and the level of it could be influenced by environmental factors, the degree of ripeness and other factors, such as type of soil, the degree of exposure to sun and rainfall [7]. Higher total phenolics in sweet potato were observed at locations with longer days and cooler temperatures [42]. Cultivation methods also have an influence on total phenolics content. Organically-cultivated sweet potatoes contain higher levels of total phenolics than conventionally-cultivated ones [42].

## 4. Conclusions

Sweet potatoes are an essential nutritious staple crop in Bangladesh. Nine varieties that were orange were used to study their nutrient composition. This study showed that the sweet potato varieties have very large genetic diversity in terms of proximate nutrients, TCC and TPC, and the extract from these cultivars may have the potential for use as natural antioxidants (carotenoids and polyphenols), which can act as a barrier to many types of cancer and other chronic diseases. This study also revealed that OFSP varieties are rich in carotenoids and also assumed to be rich in β-carotene, which is a precursor of vitamin A, and can play an important role in the alleviation of VAD in the children of developing countries. While the production of other sweet potato varieties is encouraged, the study findings suggest the consumption of OFSP to address nutrient deficiencies. Therefore, it is necessary to bring the beneficial role of OFSP consumption to the attention of the public, the medical profession, producers and consumers.

## Figures and Tables

**Table 1:** The flesh color, common and local names of the nine varieties of orange-fleshed sweet potato grown in Bangladesh. BARI, Bangladesh Agricultural Research Institute; SP, sweet potato.

Sample Number	Common Name	Flesh Color	English Name	Local Name
1	BARI SP 1	Light Orange	Sweet Potato	Tripti
2	BARI SP 2	Orange	Sweet Potato	Kamala Sundari
3	BARI SP 3	Light Orange	Sweet Potato	Doulotpuri
4	BARI SP 4	Orange	Sweet Potato	Misti Alu
5	BARI SP 5	Light Orange	Sweet Potato	Misti Alu
6	BARI SP 6	Light Orange	Sweet Potato	Lalkothi
7	BARI SP 7	Light Orange	Sweet Potato	Kalmegh
8	BARI SP 8	Orange	Sweet Potato	Misti Alu
9	BARI SP 9	Light Orange	Sweet Potato	Misti Alu

**Table 2:** Proximate composition (g/100 g of sample) of nine varieties of orange-fleshed sweet potato with peel.

OFSP ^1^ Varieties	Proximate Composition					
	Moisture	Protein	Fat	Crude Fiber	Ash	Carbohydrate
BARI SP 1	70.97 ± 0.07^c^	2.70 ± 0.93^d^	0.28 ± 0.22^b^	0.40 ± 0.12^bcd^	1.17 ± 0.02	24.50 ± 0.13^a^
BARI SP 2	71.85 ± 1.00^ac^	2.20 ± 1.05^e^	0.17 ± 0.10^d^	0.36 ± 0.02^cd^	1.19 ± 0.02	24.24 ± 1.06^a^
BARI SP 3	71.27 ± 0.12^bc^	2.41 ± 0.39^e^	0.26 ± 0.13^b^	0.48 ± 0.02^ab^	1.18 ± 0.04	24.41 ± 0.21^a^
BARI SP 4	70.95 ± 0.70^c^	5.83 ± 0.30^a^	0.30 ± 0.25^b^	0.54 ± 0.14^a^	1.29 ± 0.06	21.10 ± 0.93^c^
BARI SP 5	71.87 ± 0.38^ac^	2.36 ± 0.60^e^	0.63 ± 0.11^a^	0.35 ± 0.16^c^	1.25 ± 0.10	23.55 ± 0.51^ab^
BARI SP 6	72.96 ± 0.15^a^	1.91 ± 1.01^f^	0.24 ± 0.10^bc^	0.46 ± 0.17^ab^	1.17 ± 0.09	23.28 ± 0.05^a^
BARI SP 7	71.77 ± 0.85^ac^	2.19 ± 0.75^e^	0.25 ± 0.15^bc^	0.30 ± 0.11^d^	1.20 ± 0.04	24.29 ± 0.86^a^
BARI SP 8	71.25 ± 0.32^bc^	5.12 ± 1.26^b^	0.19 ± 0.15^cd^	0.53 ± 0.13^a^	1.31 ± 0.04	21.61 ± 0.44^c^
BARI SP 9	72.44 ± 0.38^ac^	3.66 ± 0.84^c^	0.26 ± 0.20^bc^	0.44 ± 0.16^ac^	1.26 ± 0.05	21.95 ± 0.29^b^

**^1^** OFSP = orange-fleshed sweet potato; letters in superscript indicate the statistical differences among the varieties.

**Table 3:** Total polyphenol content and total carotenoids content of selected varieties of orange-fleshed sweet potato with peel.

OFSP ^1^ Varieties	Total Polyphenol Content (mg GAE/100 g fresh weight)	Total Carotenoids Content (mg/100 g fresh weight)
BARI SP 1	104.19 ± 1.10^e^	0.55 ± 0.41^d^
BARI SP 2	114.25 ± 3.44^c^	6.38 ± 1.19^a^
BARI SP 3	106.53 ± 2.52^de^	1.09 ± 0.08^cd^
BARI SP 4	133.92 ± 4.89^a^	3.72 ± 1.11^b^
BARI SP 5	118.27 ± 1.54^bc^	2.12 ± 0.28^cd^
BARI SP 6	94.63 ± 1.40^f^	0.38 ± 0.21^d^
BARI SP 7	136.05 ± 3.53^a^	3.01 ± 0.15^b^
BARI SP 8	111.56 ± 1.50^cde^	7.24 ± 0.19^a^
BARI SP 9	117.11 ± 1.52^c^	4.61 ± 0.11^b^

**^1^** OFSP = orange-fleshed sweet potato; letters in superscript across the column indicate the statistical differences among the varieties.
